# Correlation Between the Atherogenic Index of Plasma and Major Adverse Long-Term Prognosis in Patients With Coronary Artery Disease and Stage 2–5 Chronic Kidney Disease

**DOI:** 10.31083/RCM48784

**Published:** 2026-07-17

**Authors:** Hongya Liu, Jiayi Ren, Chao Jiang, Xian Shao, Jinhua Zhao, Kangyin Chen

**Affiliations:** ^1^Tianjin Key Laboratory of Ionic-Molecular Function of Cardiovascular Disease, Department of Cardiology, Tianjin Institute of Cardiology, The Second Hospital of Tianjin Medical University, 300211 Tianjin, China; ^2^Department of Cardiology, Heart Center, Inner Mongolia People’s Hospital, 010017 Hohhot, Inner Mongolia Autonomous Region, China; ^3^Division of Nephrology, Nanfang Hospital, Southern Medical University, National Clinical Research Center for Kidney Disease, State Key Laboratory of Multi-organ Injury Prevention and Treatment, Guangdong Provincial Institute of Nephrology, Guangdong Provincial Key Laboratory of Renal Failure Research, 510515 Guangzhou, Guangdong, China

**Keywords:** coronary artery disease, chronic kidney disease, prognosis, atherogenic index of plasma

## Abstract

**Background:**

The prognostic utility of the atherogenic index of plasma (AIP) in patients with coronary artery disease (CAD) and chronic kidney disease (CKD) remains unclear. Therefore, this study aimed to evaluate the association between AIP and long-term outcomes in patients with CAD and stage 2–5 CKD.

**Methods:**

This retrospective study included 1816 patients with angiographically confirmed CAD and stage 2–5 CKD treated at a tertiary center between January 2019 and June 2023. The AIP was calculated as log10 (TG/HDL-C). The primary endpoint was major adverse cardiac and cerebrovascular events (MACCEs), including cardiac death, non-fatal myocardial infarction (MI), non-fatal stroke, and ischemia-driven revascularization. Patients were stratified into high- and low-AIP groups according to the optimal receiver operating characteristic (ROC) curve-derived cutoff value (0.148). The association between the AIP and incidence of MACCEs was primarily evaluated in the overall cohort using multivariable Cox regression. To minimize the influence of confounding and improve comparability between groups, Kaplan–Meier analysis and model performance metrics, including the C-statistic, net reclassification improvement (NRI), and integrated discrimination improvement (IDI), were also assessed.

**Results:**

MACCEs occurred in 379 patients (20.9%). High AIP independently predicted increased risk of MACCEs (adjusted hazard ratio (HR): 1.95; 95% confidence interval (CI): 1.45–2.63; *p* < 0.001). Specifically, the high-AIP group showed a significantly elevated risk of cardiac death (HR: 2.07; 95% CI: 1.13–3.80; *p *= 0.019), non-fatal MI (HR: 1.97; 95% CI: 1.02–3.78; *p *= 0.043), and ischemia-driven revascularization (HR: 2.97; 95% CI: 1.76–5.02; *p* < 0.001). Incorporation of AIP values into the established Global Registry of Acute Coronary Events (GRACE) risk model improved predictive accuracy (C-statistic: 0.624 to 0.679; NRI = 0.139; *p *= 0.01; IDI = 0.037; *p* < 0.001) with an optimal AIP cutoff of 0.148 for predicting–MACCEs.

**Conclusions:**

AIP is an independent predictor of adverse long-term outcomes in patients with CAD and stage 2–5 CKD. Integration of the AIP into existing risk models significantly enhances risk stratification and facilitates the identification of high-risk individuals for personalized management.

## 1. Introduction

Cardiovascular diseases significantly contribute to the global public health burden, with coronary artery disease (CAD) having the highest mortality rate [1]. Studies have shown that chronic kidney disease (CKD) significantly increase the risk of CAD and can exacerbate cardiovascular disease outcomes. Patients with comorbid CAD and CKD experience a notably higher incidence of major adverse cardiac and cerebrovascular events (MACCE) [2]. However, Theofilis et al. [3] found that traditional lipid indices lack predictive power when assessing cardiovascular risk in patients with CAD and CKD [4]. Renal insufficiency-induced oxidative stress, inflammation, and insulin resistance can worsen atherosclerosis and arterial damage, thereby elevating the risk of cardiovascular events and mortality [5,6]. As such, in patients with CAD combined with CKD, the higher incidence of MACCE cannot be fully explained by the isolated evaluation of traditional cardiovascular risk factors, such as hypertension, diabetes, elevated serum creatinine, or hyperlipidemia, when used as individual indices. There is thus a pressing clinical need to identify novel prognostic indicators suitable for evaluating patients with CAD combined with CKD as a means of selecting novel targets that may offer avenues for subsequent treatment.

The atherogenic index of plasma (AIP) is calculated as the base-10 logarithm of the ratio of plasma triglyceride (TG) to high-density lipoprotein cholesterol (HDL-C) levels (log_10_ (TG/HDL-C)) [7]. AIP values are negatively correlated with both lipoprotein particle diameter and the HDL-C esterification rate. Previous studies have revealed that an increase in plasma TG levels is associated with the occurrence and progression of insulin resistance [8], while the HDL-C fraction contains multiple components with anti-inflammatory and antioxidant properties. By integrating data on both TG and HDL-C levels, the AIP reflects lipid particles size and the degree of any lipid metabolism abnormalities, while also capturing information on underlying inflammatory activity and insulin resistance [9,10].

The AIP is currently used as an alternative biomarker to small dense low-density lipoprotein (sdLDL) [11], is considered an independent predictor of rapid plaque progression [12], and is employed when assessing cardiovascular disease risk. The prognostic significance of AIP has recently been validated in patients with CAD who have comorbid diabetes, hypertension, ischemic stroke, or are undergoing renal dialysis [13,14,15]. However, the association between AIP and prognosis in patients with CAD combined with CKD remains unclear. Therefore, the objective of this study was to evaluate the potential value of AIP as a predictor of a long-term adverse prognosis in patients with CAD combined with CKD.

## 2. Methods

### 2.1 Study Population and Design

**Fig. 1.**
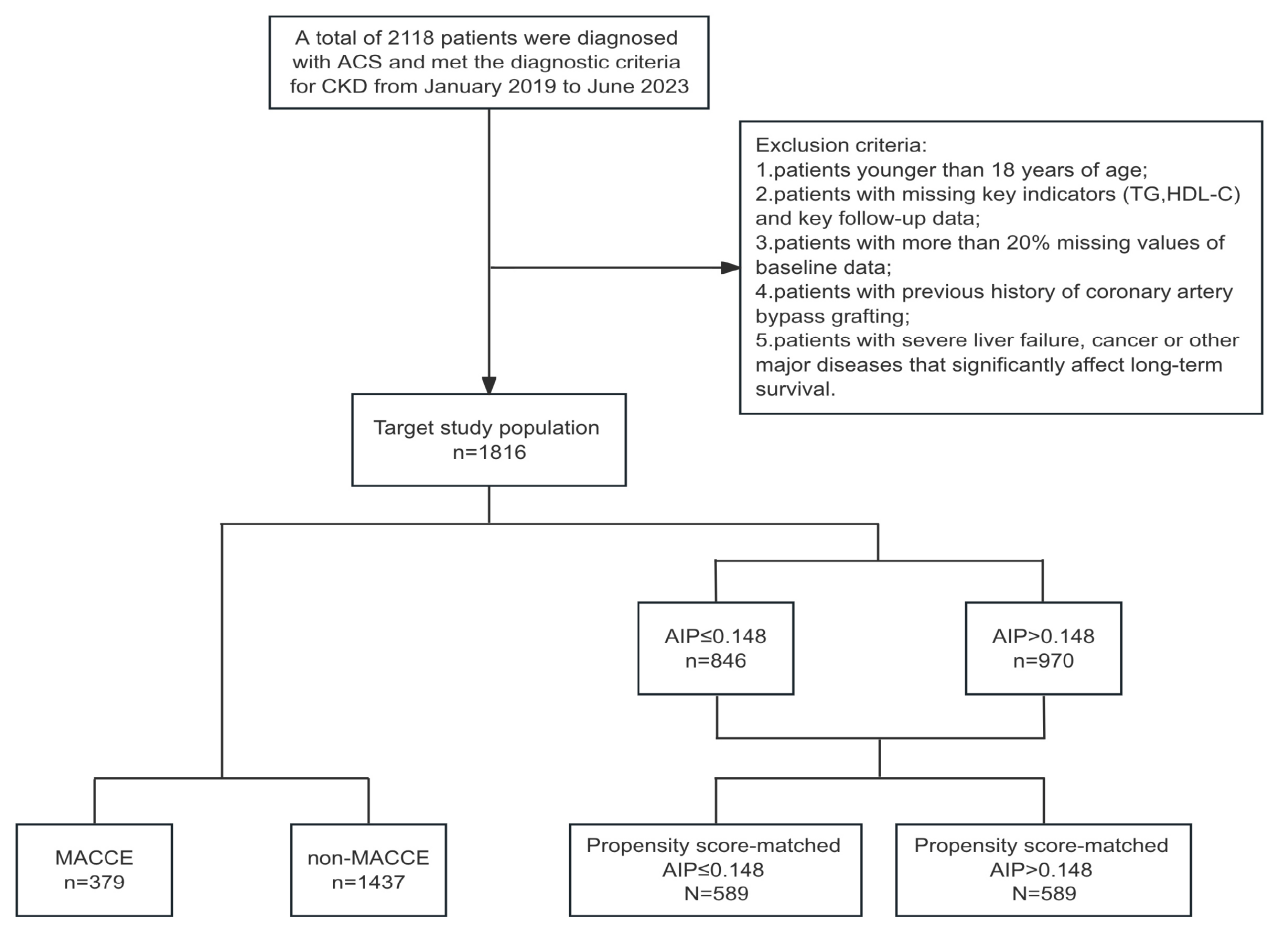
**Flowchart of the study design**. ACS, acute coronary syndrome; CKD, chronic kidney disease; TG, triglycerides; HDL-C, high-density lipoprotein cholesterol; AIP, atherogenic index of plasma; MACCE, major adverse cardiac and cerebrovascular events.

This was designed as a large single-center retrospective cohort study conducted at the Second Hospital of Tianjin Medical University, China. During the study period from January 2019 to June 2023, a total of 11,038 patients were diagnosed with CAD through coronary angiography, among whom 2118 were additionally diagnosed with Stage 2–5 CKD (combined CAD and CKD group) and were included in this study (Fig. 1). Study exclusion criteria were as follows: (1) age <18 years; (2) missing key indicators (TG or HDL-C) and essential follow-up data; (3) >20% missing baseline data; (4) a history of coronary artery bypass graft (CABG); (5) severe liver failure, cancer, or other major diseases that significantly affected their long-term survival. A total of 1816 patients met the inclusion criteria and were included in the statistical analysis. As this study involved retrospective analysis of de-identified clinical data, the requirement for written informed consent was waived by the same ethics committee. This study was conducted in accordance with the Declaration of Helsinki and was approved by the Clinical Research Ethics Committee of the Second Hospital of Tianjin Medical University (Tianjin, China). The ethics approval number is KY2023053-01.

### 2.2 Data Collection and Definitions

Relevant experimental data were obtained retrospectively from the medical records of all study subjects (missing non-critical data with <20% missing values were imputed using multiple imputation methods). The collected data included baseline demographic characteristics, medical history, laboratory parameters, coronary angiography findings, left ventricular ejection fraction (LVEF), and treatment information. Coronary angiographic findings were collected primarily to confirm the diagnosis of CAD. However, detailed angiographic variables required for standardized severity scoring were not systematically available in the retrospective dataset. Laboratory data were obtained from venous blood samples collected after an overnight fast on the day of admission. The estimated glomerular filtration rate (eGFR) was calculated using the Modification of Diet in Renal Disease (MDRD) GFR equation [16], as follows: eGFR (mL/min/1.73 m^2^) = 175 × Scr (mg/dL)^–1.234^ × age (year)^–0.179^ × (0.79 if female) mg/dL. Non-HDL-C was calculated as total cholesterol minus HDL-C. The diagnostic criteria for CKD were based on prior reports [17]: requiring the presence of at least one of the following for at least the last three months: albuminuria (AER ≥30 mg/d; ACR ≥30 mg/g), urinary sediment abnormalities; electrolyte and other abnormalities due to tubular disorders; abnormalities detected using histology; structural abnormalities detected using imaging; history of kidney transplantation; and GFR <60 mL/min/1.73 m^2^. Diagnosis with CKD (Stage 2–5) was based on patients meeting the diagnostic criteria for CKD and exhibiting an eGFR <90 mL/min/1.73 m^2^. The staging criteria for CKD were as follows: mild renal insufficiency (Stage 2): 60 mL/min/1.73 m^2^ ≤ eGFR < 90 mL/min/1.73 m^2^; moderate renal insufficiency (Stage 3): 30 mL/min/1.73 m^2^ ≤ eGFR < 60 mL/min/1.73 m^2^; severe renal insufficiency (Stage 4): 15 mL/min/1.73 m^2^ ≤ eGFR < 30 mL/min/1.73 m^2^; and renal failure (Stage 5): eGFR <15 mL/min/1.73 m^2 ^[17]. The diagnostic criteria for acute coronary syndrome (ACS) were based on established criteria. ST-segment elevation myocardial infarction (STEMI) was diagnosed when cardiac troponin (cTn) levels exceeded the 99th percentile upper limit of normal (ULN) or creatine kinase-MB (CK-MB) exceeded the 99th ULN, in conjunction with electrocardiographic evidence of convex upward ST-segment elevation. In addition, at least one of the following features was required: persistent ischemic chest pain, regional wall motion abnormalities detected by echocardiography, or abnormal findings on coronary angiography [18]. Non-STEMI (NSTEMI) was defined by elevated cTn (>99th ULN) or CK-MB (>99th ULN), together with at least one of the following: ongoing ischemic chest discomfort, new ST-segment depression or T-wave flattening/inversion on electrocardiography, segmental wall motion abnormalities on echocardiography, or abnormal coronary angiographic findings. Unstable angina (UA) was characterized by the absence of cTn elevation, the presence of ischemic chest pain, electrocardiographic changes such as transient ST-segment depression or T-wave flattening/inversion, and, in rare cases, transient ST-segment elevation consistent with vasospastic angina [19]. Stroke was diagnosed based on the incidence of cerebral infarction or cerebral hemorrhage, as determined based on the presence of characteristic clinical symptoms or imaging examinations [20]. For all additional patient- and lesion-related variables, internationally accepted standardized definitions were used to define the clinical diagnoses and clinical events.

### 2.3 Follow-Up and Clinical Endpoints

All patients included in the study cohort were monitored for a median duration of 811 days. Two well-trained doctors from the Second Hospital of Tianjin Medical University conducted all patient follow-up via telephone or outpatient visits, making use of medical history reviews, family communications, and clinical outcome analyses as appropriate. The primary endpoint of this study was the incidence of MACCE, which included a combination of cardiac death, non-fatal MI, non-fatal stroke, and ischemia-driven revascularization. Each component of MACCE was recorded as a secondary endpoint. In addition, all-cause mortality was evaluated as a secondary endpoint.

### 2.4 Statistical Analysis

All the statistical analyses were performed using SPSS Statistics, version 29.0 (IBM Corp., Armonk, NY, USA) and R software, version 4.3.1 (R Foundation for Statistical Computing, Vienna, Austria). Continuous variables were presented as mean ± standard deviation (SD) or median (interquartile range [IQR]), as appropriate, whereas categorical variables were presented as n (%). Continuous variables were compared between groups using *t*-tests or ANOVAs, while categorical variables were analyzed using Pearson χ^2^ test or Fisher’s exact test. A univariate Cox proportional hazards regression model was initially used to identify individual clinical risk factors associated with MACCE incidence. To assess the utility of AIP as an independent predictor of MACCE, the following three multivariable Cox proportional hazards regression models that considered different confounding factors were then constructed: Model 1, adjusted only for age and sex; Model 2, further adjusted for factors with a *p *value < 0.05 in univariate analyses (excluding TG and HDL-C); Model 3, further adjusted for age, hyperlipidemia, stroke history, smoking, percutaneous coronary intervention (PCI), systolic blood pressure (SBP), serum creatinine (Scr), low-density lipoprotein cholesterol (LDL-C), non-HDL-C, antiplatelet drug use, and statin use. Spearman correlation analyses were conducted to examine the relationship between the AIP and traditional cardiovascular risk factors. Receiver operating characteristic (ROC) curve analysis was additionally conducted, and the area under the curve (AUC) and the optimal cutoff value for AIP were calculated. This cutoff value was then used to divide patients into a low-AIP group and a high-AIP group. Although the sample sizes of the two groups were broadly comparable, group-size similarity does not necessarily imply balance in baseline covariates. Therefore, to reduce the influence of measured confounding and improve baseline comparability in this observational study, 1:1 propensity score matching was performed using a greedy matching algorithm with a caliper width of 0.04 of the standard deviation of the propensity score logit. The variables used for propensity score matching were selected from the multivariable model (*p *< 0.05). Event-free survival curves were plotted using the Kaplan-Meier method, and differences between groups were evaluated using the log-rank test. In subgroup analyses, age, sex, hypertension, diabetes, ACS (NSTEMI and STEMI), CKD stage, dialysis treatment status, and LDL-C levels (LDL-C ≤1.81 mmol/L vs. LDL-C >1.81 mmol/L) were assessed to determine whether the association between AIP and MACCE differed across subgroups, calculating *p* values for these interactions. In the sensitivity analysis, the robustness of the primary results was assessed by modifying the exclusion criteria to exclude patients who experienced MACCE within a short time window (30 days). In the final stage of this study, the effectiveness of AIP in predicting MACCE incidence was evaluated. Owing to the current lack of established risk scoring approaches for ACS combined with CKD, and considering the widespread application of the Global Registry of Acute Coronary Events (GRACE) risk score in predicting the clinical prognosis of patients with ACS, the value of the GRACE score as a predictor of MACCE risk was evaluated in patients with ACS combined with Stage 2–5 CKD in this study. In addition, the incremental prognostic value of AIP beyond the GRACE risk score was evaluated in patients with ACS and Stage 2–5 CKD. Specifically, the original GRACE risk score was entered as the baseline model, and AIP was then added as an additional predictor to construct an extended model. The predictive performance of the baseline and extended models was compared using the C-statistic, continuous net reclassification improvement (NRI), and integrated discrimination improvement (IDI). Continuous NRI and IDI were calculated using the “survIDINRI” R package. All result values were considered statistically significant at a two-sided *p *< 0.05.

## 3. Results

### 3.1 Baseline Characteristics of MACCE and Non-MACCE Patients

As shown in Table 1, a total of 1816 patients were included in this study. The average age of these patients was 69.7 ± 10.5 years, and 1033 (56.9%) of them were male. After a median follow-up of 811 days, 379 patients (20.9%) experienced MACCE, which included 104 cases (5.7%) of cardiac death, 85 cases (4.7%) of non-fatal MI, 128 cases (7.0%) of non-fatal stroke, 140 cases (7.7%) of ischemia-driven revascularization, and 198 cases (10.9%) of all-cause mortality. In the overall cohort, the median AIP was 0.18 (IQR, −0.01 to 0.36). To further characterize its distribution, a histogram/density plot of AIP values in the study population was prepared (**Supplementary Fig. 1**). Patients who experienced MACCE were more likely to be male and to have a history of MI, hypertension, diabetes, hyperlipidemia, anemia, and alcohol consumption. With respect to laboratory and clinical parameters, patients who experienced MACCE also exhibited lower RBC, Hb, HDL-C, eGFR, and LVEF values, as well as higher urea, Scr, UA, TG, FPG, TnI, CK-MB, and NT-proBNP values. In terms of treatment, significant differences between the two groups of patients were noted in the use of beta-blockers, insulin therapy, and dialysis therapy. No other significant differences were noted between the two groups (*p *> 0.05).

**Table 1.** **Baseline characteristics of the study population stratified by MACCE**.

Parameter		Total patients (n = 1816)	non-MACCE (n = 1437)	MACCE (n = 379)	*p* value
General characteristics					
	Age (years)	69.7 ± 10.5	69.8 ± 10.3	69.4 ± 11.2	0.538
	Males, n (%)	1033 (56.9%)	794 (55.3%)	239 (63.1%)	0.008
Medical history and risk factors, n (%)					
	Previous MI	257 (14.2%)	180 (12.5%)	77 (20.3%)	<0.001
	Previous AF	123 (6.8%)	89 (6.2%)	34 (9.0%)	0.072
	Stroke history	399 (22.0%)	307 (21.4%)	92 (24.3%)	0.251
	Anemia	103 (5.7%)	72 (5.0%)	31 (8.2%)	0.025
	Peripheral arterial disease	51 (2.8%)	38 (2.6%)	13 (3.4%)	0.516
CKD stage					<0.001
	Stage 2	1055 (58.1%)	888 (61.8%)	167 (44.1%)	
	Stage 3	402 (22.1%)	301 (20.9%)	101 (26.6%)	
	Stage 4	118 (6.5%)	81 (5.6%)	37 (9.8%)	
	Stage 5	241 (13.3%)	167 (11.6%)	74 (19.5%)	
Hypertension		1534 (84.5%)	1195 (83.2%)	339 (89.4%)	0.003
Diabetes		718 (39.5%)	518 (36.0%)	200 (52.8%)	<0.001
Hyperlipidemia		215 (11.8%)	158 (11.0%)	57 (15.0%)	0.038
Smoking		565 (31.1%)	442 (30.8%)	123 (32.5%)	0.567
Drinking		426 (23.5%)	358 (24.9%)	68 (17.9%)	0.005
Clinical presentation, n (%)					
	MI	691 (38.1%)	507 (35.3%)	184 (48.5%)	<0.001
	PCI	1420 (78.2%)	1132 (78.8%)	288 (76.0%)	0.272
Physical & Laboratory Examination					
	SBP (mmHg)	138 (124, 154)	138 (124, 154)	140 (123, 155)	0.306
	DBP (mmHg)	79 (71.0, 88.0)	79 (71.0, 88.0)	80 (71.0, 88.0)	0.786
	HR (bpm)	74.9 ± 16.2	74.6 ± 16.0	76.0 ± 16.8	0.128
	LVEF (%)	59 (50.0, 63.0)	60 (52.0, 64.0)	57 (44.0, 62.0)	<0.001
	Hb (g/L)	127 ± 22.5	129 ± 21.6	122 ± 24.7	<0.001
	PLT (×10^9^/L)	213 (177, 255)	214 (180, 254)	207 (166, 258)	0.164
	Urea (mmol/L)	7.90 (6.30, 10.8)	7.70 (6.20, 10.0)	9.10 (6.80, 14.0)	<0.001
	Scr (μmol/L)	111 (95.2, 175)	108 (92.8, 160)	136 (102, 268)	<0.001
	eGFR (mL/min/1.73 m^2^)	67.8 (39.1, 81.5)	70.6 (43.9, 82.3)	54.6 (24.8, 77.3)	<0.001
	UA (μmol/L)	385 (317, 467)	382 (316, 459)	401 (324, 491)	0.036
	TC (mmol/L)	4.41 (3.70, 5.20)	4.42 (3.73, 5.24)	4.33 (3.60, 5.10)	0.140
	TG (mmol/L)	1.50 (1.10, 2.10)	1.45 (1.06, 2.06)	1.66 (1.21, 2.22)	<0.001
	HDL-C (mmol/L)	1.02 (0.86, 1.20)	1.04 (0.88, 1.23)	0.92 (0.77, 1.10)	<0.001
	LDL-C (mmol/L)	2.78 (2.21, 3.46)	2.80 (2.22, 3.47)	2.71 (2.18, 3.37)	0.477
	non-HDL-C (mmol/L)	3.37 (2.65, 4.13)	3.37 (2.65, 4.13)	3.38 (2.66, 4.14)	0.876
	FPG (mmol/L)	7.00 (5.70, 9.33)	6.80 (5.70, 9.00)	7.90 (6.30, 11.6)	<0.001
	TnI (μg/L)	0.25 (0.01, 8.98)	0.16 (0.01, 9.46)	0.80 (0.03, 7.85)	0.006
	CK-MB (u/L)	14.3 (10.3, 24.8)	13.8 (10.2, 23.9)	15.9 (11.2, 29.7)	0.012
	NT-proBNP (pg/mL)	810 (189, 3399)	595 (152, 2662)	1985 (502, 6716)	<0.001
	AIP	0.18 (−0.01, 0.36)	0.14 (−0.03, 0.34)	0.25 (0.10, 0.43)	<0.001
Discharge Medications, n (%)					
	Antiplatelet drugs	1652 (91.0%)	1302 (90.6%)	350 (92.3%)	0.341
	Statins	1653 (91.0%)	1309 (91.1%)	344 (90.8%)	0.922
	Beta blockers	1020 (56.2%)	778 (54.1%)	242 (63.9%)	0.001
	ACEI or ARB	431 (23.7%)	354 (24.6%)	77 (20.3%)	0.091
	CCB	795 (43.8%)	615 (42.8%)	180 (47.5%)	0.114
	Oral hypoglycemic agents	467 (25.7%)	357 (24.8%)	110 (29.0%)	0.112
	Insulin therapy	459 (25.3%)	346 (24.1%)	113 (29.8%)	0.026
	Dialysis therapy	36 (2.0%)	23 (1.6%)	13 (3.4%)	0.030

Note: Values are presented as n (%), mean ± SD, or median (IQR), as appropriate. MACCE, major adverse cardiac and cerebrovascular events; MI, myocardial infarction; AF, atrial fibrillation; PCI, percutaneous coronary intervention; SBP, systolic blood pressure; DBP, diastolic blood pressure; HR, heart rate; LVEF, left ventricular ejection fraction; Hb, hemoglobin; PLT, platelet; urea, carbamide; Scr, serum creatinine; eGFR, estimated glomerular filtration rate; UA, uric acid; TC, total cholesterol; LDL-C, low-density lipoprotein cholesterol; FPG, fasting plasma glucose; TnI, troponin I; CK-MB, creatine kinase-MB; NT-proBNP, N-terminal pro-brain natriuretic peptide; ACEI, angiotensin-converting enzyme inhibitors; ARB, angiotensin receptor blockers; CCB, calcium channel blocker.

### 3.2 Association Between AIP Values and MACCE Incidence

In the univariate Cox regression analysis (**Supplementary Table 1**), AIP values were significantly positively correlated with MACCE incidence (HR = 3.49; 95% CI 2.47–4.93; *p *< 0.001). To test the robustness of this association, three multivariable Cox regression models were established incorporating a series of confounding factors (Table 2). In Model 1, after adjusting for age and sex, the relationship between AIP values and MACCE incidence remained significant (adjusted HR = 3.74; 95% CI 2.61–5.36; *p *< 0.001). In Model 2, all significant variables from the univariate analysis (*p* < 0.05) were introduced, and the AIP value was still associated with an increased risk of MACCE (adjusted HR = 2.78; 95% CI 1.92–4.02; *p *< 0.001). The association remained intact with further adjustment in Model 3 (adjusted HR = 3.02; 95% CI 1.83–4.97; *p *< 0.001), supporting the existence of a significant correlation between an increased AIP and a greater risk of MACCE.

**Table 2.** **Multivariable Cox regression models for the associations between AIP and MACCE**.

Variable	HR	95% CI	*p* value
Model 1	3.74	2.61–5.36	**<**0.001
Model 2	2.78	1.92–4.02	**<**0.001
Model 3	3.02	1.83–4.97	**<**0.001

Note: HR, hazard ratio; CI, confidence interval. Model 1: Adjusted for age and sex. Model 2: Adjusted for sex, previous MI, hypertension, diabetes, CKD stage, anemia, MI, HR, Hb, urea, eGFR, UA, FPG, TnI, NT-proBNP, LVEF, beta blocker, ACEI or ARB, oral hypoglycemic agents, and dialysis therapy. Model 3: Adjusted for sex, age, previous MI, hypertension, diabetes, hyperlipidemia, stroke history, CKD stage, smoking, anemia, MI, PCI, SBP, HR, Hb, urea, Scr, eGFR, UA, LDL-C, non-HDL-C, FPG, TnI, NT-proBNP, LVEF, antiplatelet drugs, statins, beta blockers, ACEI or ARB, oral hypoglycemic agents, and dialysis therapy*.*

### 3.3 Correlations Between the AIP Value and Cardiovascular Risk Factors

As shown in Table 3, Spearman correlation analyses revealed that AIP values were positively correlated with levels of TC (r = 0.188), LDL-C (r = 0.245), FPG (r = 0.199), urea (r = 0.142), Scr (r = 0.166), and UA (r = 0.202) (*p *< 0.001) while they were negatively correlated with eGFR (r = –0.142) (*p *< 0.001). AIP was not significantly associated with SBP or LVEF (*p *> 0.05).

**Table 3.** **Correlations between the AIP score and other variables**.

Variable	Spearman correlation coefficient (r)	*p* value
SBP	0.039	0.096
TC	0.188	<0.001
LDL-C	0.245	<0.001
FPG	0.199	<0.001
Urea	0.142	<0.001
Scr	0.166	<0.001
eGFR	–0.142	<0.001
UA	0.202	<0.001
LVEF	0.008	0.749

Note: Values are Spearman correlation coefficients.

### 3.4 Identification of an Optimal AIP Cutoff Value for MACCE Prediction

**Fig. 2.**
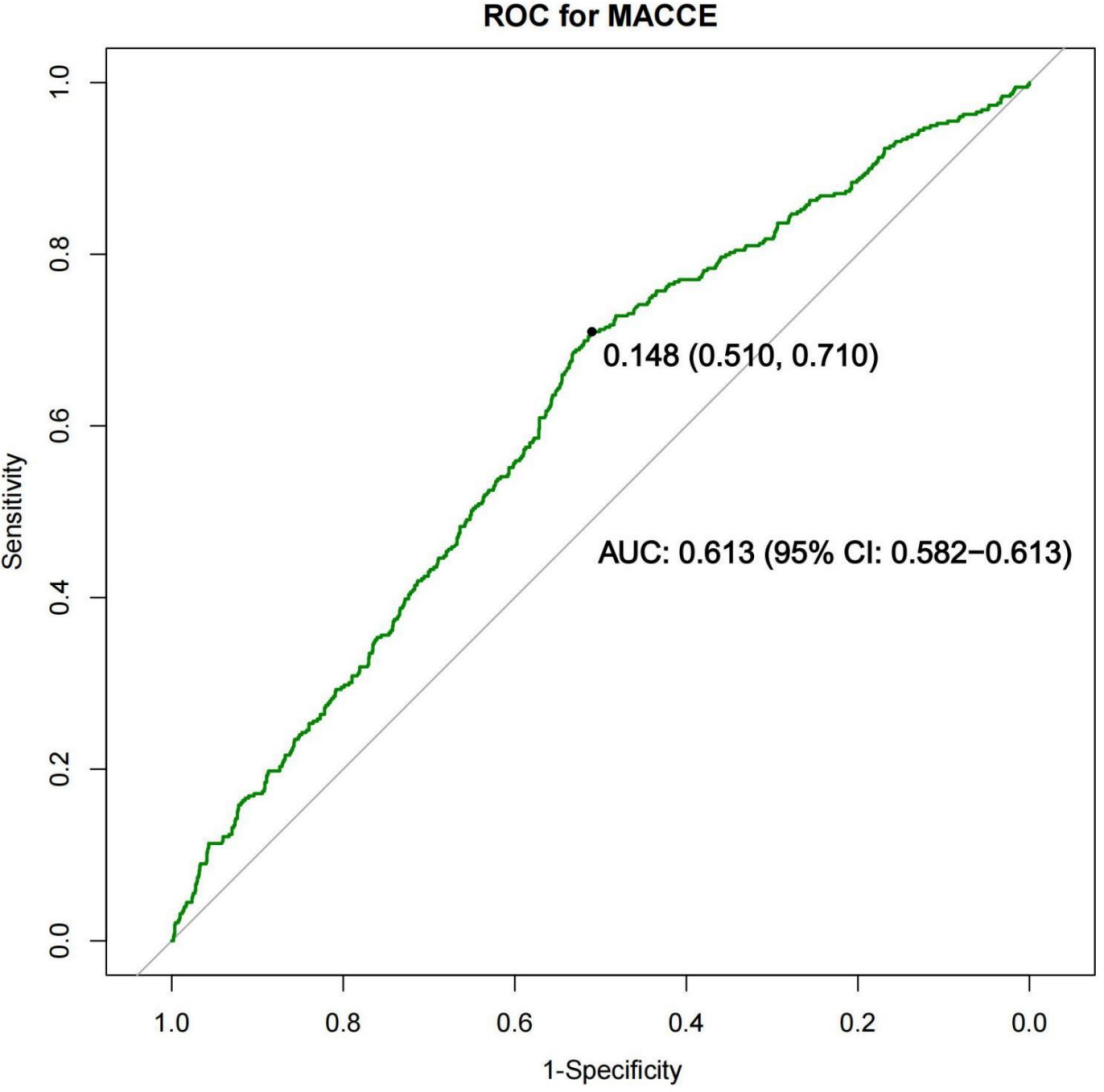
**ROC curve for the prediction of MACCE incidence based on AIP values, with the optimal AIP cutoff value being identified**. AUC, area under the curve.

Based on the ROC analysis (Fig. 2), an optimal cutoff value for predicting MACCE incidence based on AIP values was identified as 0.148, with an area under the curve (AUC) of 0.613 (95% CI 0.582–0.613), indicating a modest discriminatory ability for predicting MACCE incidence in patients with CAD and CKD.

### 3.5 Baseline Characteristics After Propensity Score Matching

After describing baseline characteristics according to MACCE status, we next categorized patients by the ROC-derived AIP cutoff for propensity score matching analyses. To improve baseline comparability between the two groups and to further mitigate the potential influence of measured confounding factors on the results of this study, patients were next divided into a high-AIP group (AIP >0.148) and a low-AIP group (AIP ≤0.148) based on the optimal AIP cutoff value identified above. Next, 1:1 propensity score matching was performed for these two groups, yielding a total of 589 matched pairs.

**Fig. 3.**
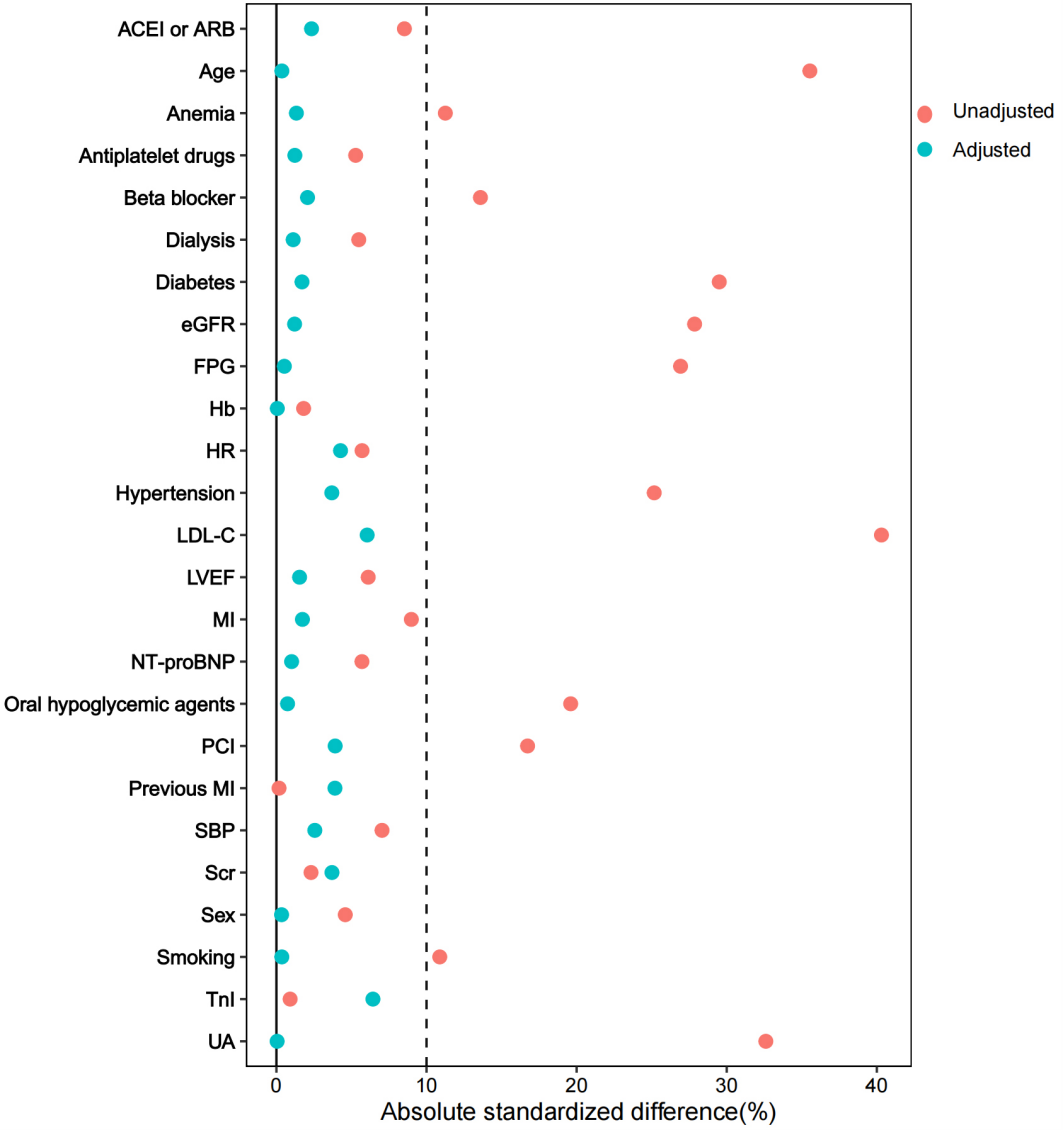
**Absolute standardized differences for the baseline covariates before and after propensity score matching**. HR, heart rate.

After matching, no significant differences in the baseline characteristics were observed between the two groups, and all standardized mean differences were <0.1, indicating adequate covariate balance (Fig. 3).

### 3.6 Cox Analysis and Kaplan-Meier Curves for the Primary and Secondary Endpoints (Propensity Score-Matched Population)

**Fig. 4.**
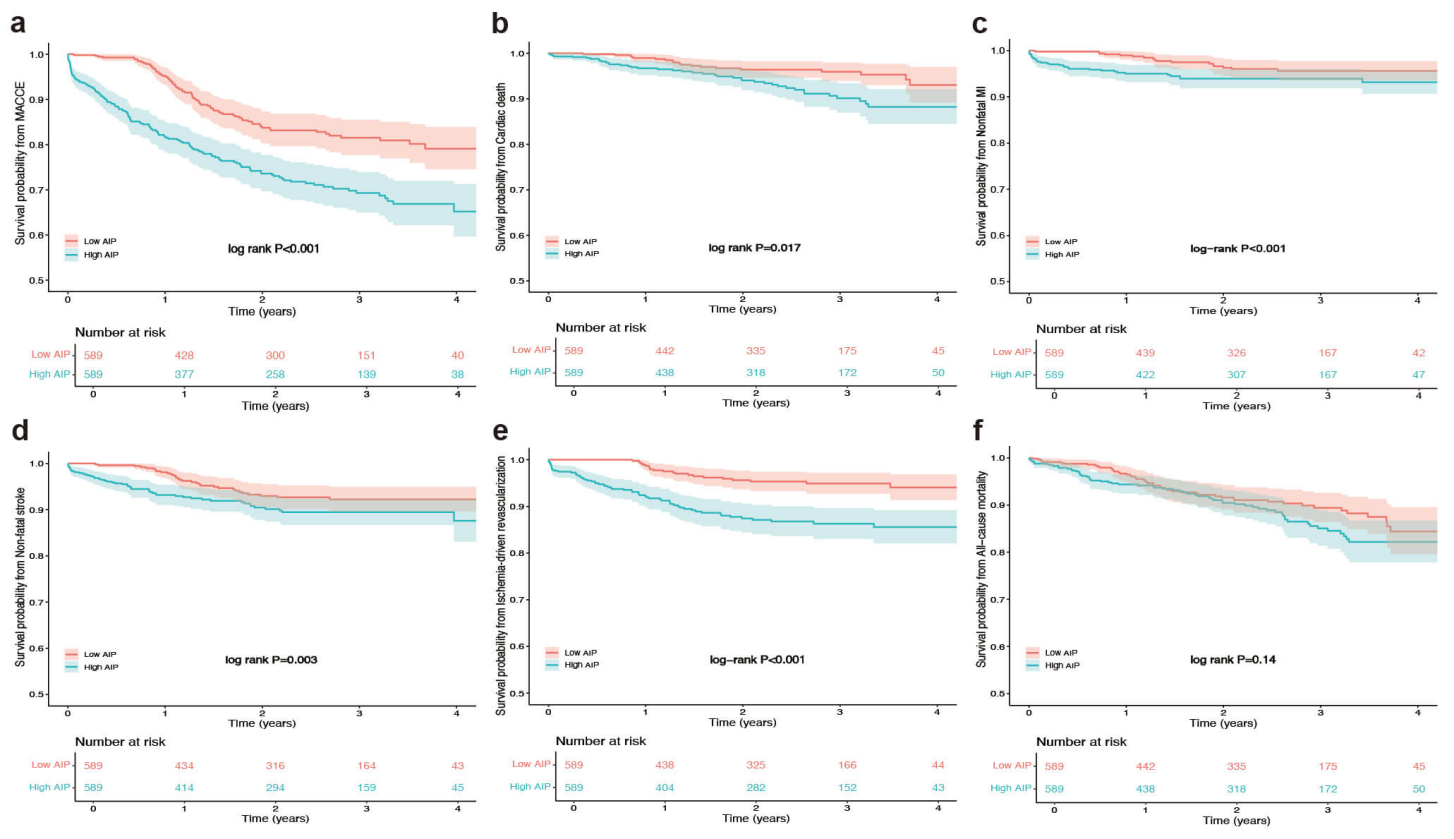
**Kaplan-Meier survival curves comparing the low- and high-AIP groups for MACCE and the secondary endpoints**. (a) MACCE; (b) Cardiac death; (c) Non-fatal myocardial infarction (MI); (d) Non-fatal stroke; (e) Ischemia-driven revascularization; (f) All-cause mortality.

To further validate the association between elevated AIP values and MACCE incidence noted in the overall cohort, these analyses were repeated in the propensity score-matched cohort with improved baseline comparability. As shown in Table 4, patients in the high-AIP group remained at a significantly increased risk of MACCE compared with those in the low-AIP group after matching (147 [25.0%] vs. 78 [13.2%]; adjusted HR = 1.95; 95% CI 1.45–2.63; *p *< 0.001). With respect to secondary endpoints, compared to the low-AIP group, patients in the high-AIP group exhibited a significantly increased risk of cardiac death (40 (6.79%) vs. 19 (3.23%); adjusted HR = 2.07; 95% CI 1.13–3.80; *p* = 0.019), non-fatal MI (32 (5.43%) vs. 17 (2.89%); adjusted HR = 1.97; 95% CI 1.02–3.78; *p* = 0.043), and ischemia-driven revascularization (63 (10.7%) vs. 21 (3.57%); adjusted HR = 2.97; 95% CI 1.76–5.02; *p *< 0.001). However, there were no significant differences in the incidence of non-fatal stroke (50 (8.49%) vs. 31 (5.26%); adjusted HR = 1.37; 95% CI 0.83–2.26; *p* = 0.2) or all-cause mortality (64 (10.9%) vs. 50 (8.49%); adjusted HR = 1.21; 95% CI 0.80–1.82; *p* = 0.4) between these two groups. To conduct an overall survival analysis, the Kaplan-Meier survival curves for the two groups of patients were compared using the log-rank test (Fig. 4). Compared to the low-AIP group, the high-AIP group exhibited significantly higher incidence of MACCE, cardiac death, non-fatal MI, non-fatal stroke, and ischemia-driven revascularization (log-rank *p* < 0.05). No significant difference was observed for all-cause mortality (log-rank *p* = 0.14).

**Table 4.** **Multivariable Cox regression analyses of clinical endpoints in the propensity score-matched cohort**.

Adverse events	Propensity score-matched population				
	Low-AIP (n = 589)	High-AIP (n = 589)	*p* value	Adjusted HR^a^ (95% CI)	*p *value
MACCE	78 (13.2%)	147 (25.0%)	<0.001	1.95 (1.45–2.63)	<0.001
Cardiac death	19 (3.23%)	40 (6.79%)	0.008	2.07 (1.13–3.80)	0.019
Non-fatal MI	17 (2.89%)	32 (5.43%)	0.041	1.97 (1.02–3.78)	0.043
Non-fatal stroke	31 (5.26%)	50 (8.49%)	0.038	1.37 (0.83–2.26)	0.2
Ischemia-driven revascularization	21 (3.57%)	63 (10.7%)	<0.001	2.97 (1.76–5.02)	<0.001
All-cause mortality	50 (8.49%)	64 (10.9%)	0.2	1.21 (0.80–1.82)	0.4

Note: Values are presented as n (%) unless otherwise indicated. Values in parentheses are 95% CIs. HR, hazard ratio; CI, confidence interval; AIP, atherogenic index of plasma. Adjusted HR^a^ were adjusted for sex, age, previous MI, hypertension, diabetes, hyperlipidemia, stroke history, CKD stage, smoking, anemia, MI, PCI, SBP, HR, Hb, urea, Scr, eGFR, UA, LDL-C, non-HDL-C, FPG, TnI, NT-proBNP, LVEF, antiplatelet drugs, statins, beta blockers, ACEI or ARB, oral hypoglycemic agents, and dialysis therapy.

### 3.7 Subgroup Analysis

**Fig. 5.**
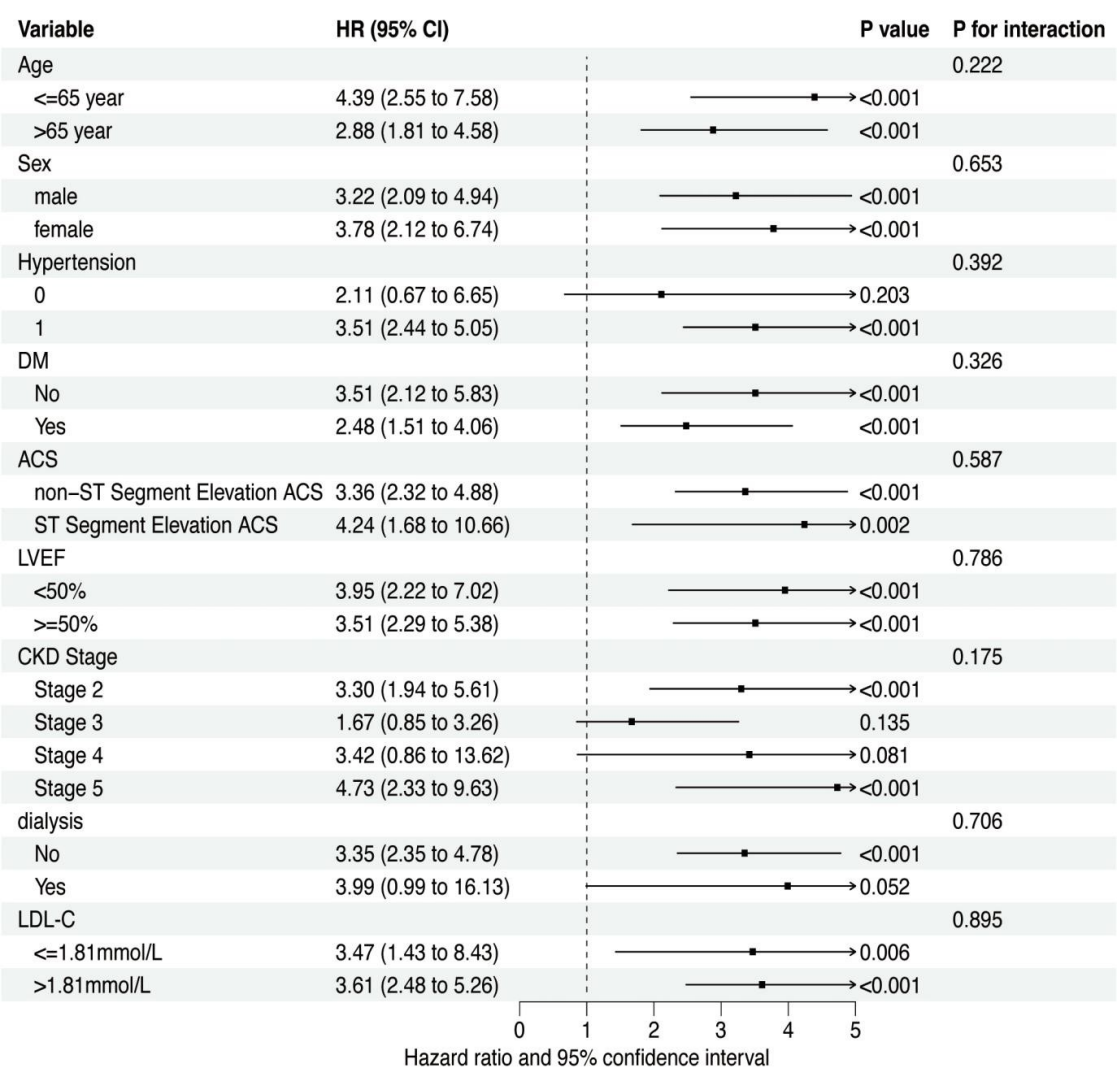
**Subgroup analysis of the associations between AIP and MACCE risk for patients stratified according to the indicated baseline characteristics**. HR, hazard ratio.

Subgroup analyses (Fig. 5) were next used to further interrogate the correlation between AIP values and MACCE risk. Among the selected subgroups, including age (≤65 vs. >65 years), sex (male vs. female), hypertension (yes vs. no), diabetes (yes vs. no), ACS (NSTEMI vs. STEMI), CKD stage (Stage 2, Stage 3, Stage 4, Stage 5), dialysis therapy (yes vs. no), and LDL-C (≤1.81 mmol/L vs. >1.81 mmol/L), the relationship between AIP values and the primary outcome remained consistent, with no significant interactions (all *p*-values for interaction > 0.05).

Because the Stage 4 CKD subgroup was relatively small, we performed an additional sensitivity analysis in the overall cohort by combining Stage 4 and Stage 5 CKD into an advanced CKD subgroup. After this regrouping, elevated AIP remained significantly associated with MACCE in both Stage 2–3 CKD (adjusted HR 1.98, 95% CI 1.47–2.66; *p* < 0.001) and Stage 4–5 CKD (adjusted HR 2.92, 95% CI 1.68–5.08; *p* < 0.001), with no statistically significant interaction (*p* for interaction = 0.149) (**Supplementary Tables 2 and 3**). When CKD stage was additionally treated as an ordinal variable, no significant interaction trend was observed (interaction HR 1.08, 95% CI 0.87–1.34; *p* = 0.481) (**Supplementary Table 4**). After excluding patients who experienced MACCE within 30 days, the results remained directionally consistent in Stage 2–3 CKD (adjusted HR 1.46, 95% CI 1.06–2.01; *p* = 0.019) and Stage 4–5 CKD (adjusted HR 2.53, 95% CI 1.41–4.54; *p *= 0.002) (**Supplementary Table 5**), again without significant interaction (*p* for interaction = 0.110). Similarly, the ordinal interaction analysis after this 30-day MACCE exclusion remained non-significant (interaction HR 1.11, 95% CI 0.89–1.38; *p* = 0.359) (**Supplementary Table 6**).

### 3.8 Sensitivity Analysis

In order to evaluate the reliability of these findings, a sensitivity analysis was conducted by excluding 103 patients who experienced MACCE within 30 days and reanalyzing the remaining cohort. In both univariate analyses and multivariate Cox regression analyses using adjusted Model 3, the positive correlation between AIP values and risk of MACCE remained significant (adjusted HR = 1.98; 95% CI 1.16–3.41; *p* = 0.013) (**Supplementary Table 7**).

### 3.9 Predictive Value of AIP for MACCE

The value of the AIP as a predictor of MACCE incidence was further examined in the context of established risk scoring models. When the AIP score was incorporated into the GRACE risk score, an improvement in the predictive ability of the GRACE score was observed (the C-index increased from 0.624 to 0.679, *p *< 0.001). This enhancement effect was also assessed using the NRI (NRI = 0.139, 95% CI 0.053–0.199, *p* = 0.01) and IDI (IDI = 0.037; 95% CI 0.015–0.060; *p* < 0.001), and the AIP exhibited superior performance in predicting the risk of MACCE (Table 5).

**Table 5.** **Incremental predictive value and reclassification statistics for the AIP value as a predictor of MACCE incidence**.

	C-statistic (95% CI)	*p* value	Continuous NRI (95% CI)	*p* value	IDI (95% CI)	*p* value
GRACE risk score	0.624 (0.595–0.653)	Ref		Ref		Ref
GRACE risk score + AIP value	0.679 (0.654–0.704)	<0.001	0.139 (0.053–0.199)	0.01	0.037 (0.015–0.060)	<0.001

Note: Values in parentheses are 95% CIs. Ref indicates the reference model. Ref, reference; NRI, net reclassification improvement; IDI, integrated discrimination improvement; GRACE, Global Registry of Acute Coronary Events; AIP, atherogenic index of plasma.

## 4. Discussion

In this retrospective cohort of patients with CAD and Stage 2–5 CKD, we found that AIP was independently associated with long-term MACCE risk. In addition, adding AIP to the GRACE score improved risk discrimination, suggesting that AIP may serve as a simple adjunctive marker for baseline risk stratification in this high-risk population.

CAD and CKD share multiple pathophysiological pathways, including oxidative stress, chronic inflammation, endothelial dysfunction, vascular calcification, and metabolic derangement [21,22,23,24]. In this setting, conventional lipid parameters widely used in cardiovascular risk assessment, including LDL-C, HDL-C, TC, and TG levels, may not fully reflect cardiovascular risk [25,26,27,28,29]. This is particularly relevant in patients with CKD, in whom reverse epidemiology and malnutrition-inflammation-related metabolic alterations may attenuate the prognostic value of isolated lipid measurements [30,31,32]. Consistent with this concept, none of the individual lipid parameters in our cohort showed a significant association with MACCE risk, whereas the AIP, which integrates TG and HDL-C into a composite index, remained independently associated with adverse outcomes. This finding supports the notion that a composite lipid-metabolic marker may better reflect the atherogenic and inflammatory milieu present in patients with coexisting CAD and CKD.

The AIP has been proposed as a composite lipid-related biomarker that is linked to lipoprotein particle characteristics, insulin resistance, and inflammatory activity [10,33,34]. In our study, higher AIP values were consistently associated with MACCE incidence across unadjusted and multivariable models, suggesting that the prognostic information carried by AIP is not fully explained by conventional cardiovascular risk factors alone. Moreover, AIP was correlated with several adverse metabolic and renal-risk profilesAIP value, including TC, LDL-C, FPG, urea, Scr, eGFR, and UA, further supporting its role as an integrated marker of cardiometabolic risk burden.

ROC analysis identified an AIP value of 0.148 as a data-derived cutoff for discriminating between patients according to MACCE risk in this cohort. When AIP was analyzed categorically using this threshold, elevated AIP remained associated with higher risks of MACCE, cardiac death, non-fatal MI, and ischemia-driven revascularization after propensity score matching and multivariable adjustment, supporting the robustness of the main findings. In contrast, the association between AIP values and non-fatal stroke incidence was not significant under adjusted Cox models, despite a difference having been observed in an initial unadjusted Kaplan-Meier analysis, potentially reflecting differences in covariate adjustment and limited statistical power for this endpoint. As such, this threshold AIP value of 0.148 should be interpreted as a cohort-derived stratification threshold rather than a universally established clinical cutoff.

An additional clinically relevant finding of this study was the observation that incorporating AIP values into the GRACE score improved discrimination and reclassification for MACCE risk. Given that the GRACE score was developed primarily for patients with ACS and was not specifically designed for individuals with concomitant renal dysfunction, this improvement suggests that AIP may capture risk information not fully represented in conventional clinical risk models [35,36]. In this context, AIP may be useful as an adjunctive marker to refine baseline risk assessment, particularly in patients with ACS and coexisting CKD, although external validation in larger independent cohorts remains necessary.

Although AIP has been widely studied in cardiovascular disease, our findings extend the literature in several respects. We specifically examined patients with CAD and Stage 2–5 CKD, a high-risk and metabolically complex population in whom conventional lipid measures may have limited prognostic value. Moreover, the association between elevated AIP and MACCE remained directionally consistent across analyses with different approaches to confounding control, including multivariable adjustment and propensity score matching, as well as across subgroup and sensitivity analyses, further supporting the robustness of the findings.

### Study Strengths and Limitations

This study has several strengths. First, we specifically focused on patients with CAD and Stage 2–5 CKD, a clinically high-risk population in whom conventional lipid parameters may have limited prognostic utility. Second, in addition to examining the association between AIP and long-term MACCE incidence, we evaluated its incremental prognostic value over the GRACE score. Moreover, the consistency of our findings across multivariable adjustment, propensity score matching, subgroup analyses, and sensitivity analyses strengthens the internal consistency of this study.

Several limitations should be acknowledged. First, this was a single-center retrospective study, and residual confounding cannot be completely excluded despite multivariable adjustment and propensity score matching. Second, AIP was measured only once at admission, without serial follow-up assessments; therefore, the present findings should be interpreted as reflecting the prognostic value of baseline AIP rather than its dynamic variation over time. Third, detailed longitudinal data on treatment exposure during follow-up were not systematically available. In addition, we did not have access to more granular information on coronary anatomical severity, BMI, long-term glycemic control as reflected by HbA1c, and other clinically relevant covariates. These factors may have introduced additional residual confounding during follow-up. Finally, although the AIP cutoff of 0.148 was associated with risk stratification in this cohort, it should be regarded as a cohort-derived threshold and requires external validation. Despite these limitations, the AIP is a simple index that can be readily derived from routine lipid testing results and may have potential value for baseline risk stratification in this high-risk population with coexisting CKD and CAD. Future prospective multicenter studies with serial biomarker measurements and more detailed longitudinal clinical data are warranted to further validate these findings.

## 5. Conclusions

In patients with CAD and Stage 2–5 CKD, higher AIP values were independently associated with an increased risk of long-term MACCE incidence. A cohort-derived AIP threshold of 0.148 identified a subgroup at higher MACCE risk, and incorporation of AIP values into the GRACE score improved risk discrimination. These findings suggest that AIP may be a simple and clinically accessible adjunct for baseline risk stratification in patients with comorbid CAD and CKD, although prospective multicenter validation is warranted before broader clinical implementation.

## Data Availability

All datasets generated and analyzed during the current study are freely available to editors, reviewers, and readers without restriction, provided that such sharing complies with applicable legal and ethical standards. Interested parties may request access by contacting the first author, Dr. Hongya Liu, at Drhongya@163.com.
