# Supplementary material for: Correlation Between the Atherogenic Index of Plasma and Major Adverse Long-Term Prognosis in Patients With Coronary Artery Disease and Stage 2–5 Chronic Kidney Disease
Source: Rev Cardiovasc Med. 2026 Jul 17;27(7):48784. doi: 10.31083/RCM48784 (PMC13419954; doi:10.31083/RCM48784)
Supplement: Supplementary file 1 [file 2153-8174-27-7-48784-s1.zip › Supplementary Material.docx]

**
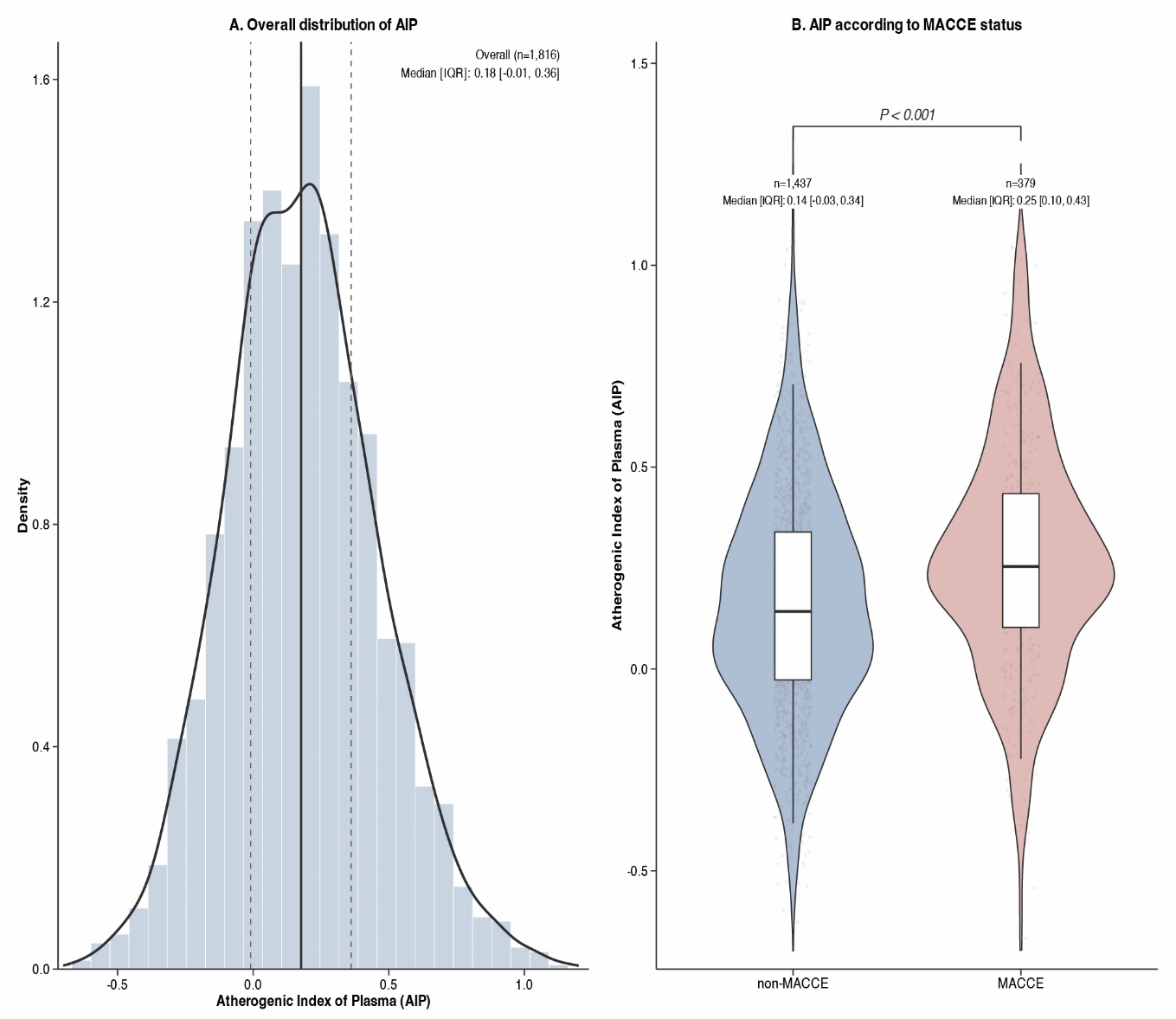
**

**Supplementary Fig. 1.** Distribution Characteristics of the Atherogenic Index of Plasma in the Study Population and by MACCE Status. MACCE, major adverse cardiac and cerebrovascular events.

**Supplementary Table 1.** Cox regression models for the associations between AIP and MACCE.

| **Characteristic** | **Univariable** | | | | **Multivariate** | | |
| --- | --- | --- | --- | --- | --- | --- | --- |
|  | **N** | **HR** | **95% CI** | ***P* value** | **HR** | **95% CI** | ***P* value** |
| Sex | 1,816 | 0.72 | 0.59, 0.89 | 0.002 | 0.76 | 0.59, 0.96 | **0.022** |
| Age | 1,816 | 1.00 | 0.99, 1.01 | 0.4 |  |  |  |
| Previous MI | 1,816 | 1.60 | 1.24, 2.05 | <0.001 | 1.36 | 1.04, 1.77 | **0.024** |
| Previous AF | 1,816 | 1.31 | 0.92, 1.86 | 0.13 |  |  |  |
| Hypertension | 1,816 | 1.56 | 1.12, 2.17 | 0.008 | 1.37 | 0.98, 1.92 | 0.066 |
| Diabetes | 1,816 | 1.91 | 1.56, 2.34 | <0.001 | 1.53 | 1.17, 2.00 | **0.002** |
| Hyperlipidemia | 1,816 | 1.10 | 0.83, 1.46 | 0.5 |  |  |  |
| Stroke History | 1,816 | 1.23 | 0.97, 1.56 | 0.082 |  |  |  |
| Peripheral arterial disease | 1,816 | 1.38 | 0.79, 2.39 | 0.3 |  |  |  |
| CKD Stage | 1,816 |  |  |  |  |  |  |
| G2 |  | — | — |  | — | — |  |
| G3 |  | 1.86 | 1.45, 2.38 | <0.001 | 1.22 | 0.73, 2.02 | 0.4 |
| G4 |  | 2.57 | 1.80, 3.67 | <0.001 | 1.13 | 0.48, 2.63 | 0.8 |
| G5 |  | 2.44 | 1.85, 3.20 | <0.001 | 1.43 | 0.50, 4.15 | 0.5 |
| Anemia | 1,816 | 2.56 | 1.77, 3.70 | <0.001 | 1.27 | 0.84, 1.92 | 0.3 |
| Smoking | 1,816 | 1.14 | 0.92, 1.41 | 0.2 |  |  |  |
| Drinking | 1,816 | 0.87 | 0.67, 1.14 | 0.3 |  |  |  |
| MI | 1,816 | 1.84 | 1.51, 2.26 | <0.001 | 1.39 | 1.09, 1.77 | **0.009** |
| PCI | 1,816 | 1.23 | 0.97, 1.55 | 0.093 |  |  |  |
| SBP | 1,816 | 1.00 | 1.00, 1.00 | 0.8 |  |  |  |
| DBP | 1,816 | 1.00 | 0.99, 1.00 | 0.4 |  |  |  |
| HR | 1,816 | 1.01 | 1.00, 1.01 | 0.014 | 1.00 | 0.99, 1.00 | 0.2 |
| Hb | 1,816 | 0.99 | 0.98, 0.99 | <0.001 | 0.99 | 0.99, 1.00 | **0.006** |
| PLT | 1,816 | 1.00 | 1.00, 1.00 | >0.9 |  |  |  |
| Urea | 1,816 | 1.06 | 1.04, 1.07 | <0.001 | 1.00 | 0.98, 1.02 | >0.9 |
| Scr | 1,816 | 1.00 | 1.00, 1.00 | 0.3 |  |  |  |
| eGFR | 1,816 | 0.99 | 0.98, 0.99 | <0.001 | 1.00 | 0.98, 1.01 | 0.8 |
| UA | 1,816 | 1.00 | 1.00, 1.00 | 0.026 | 1.00 | 1.00, 1.00 | 0.8 |
| TC | 1,816 | 0.93 | 0.85, 1.02 | 0.12 | 1.45 | 0.83, 2.55 | 0.2 |
| TG | 1,816 | 1.16 | 1.08, 1.25 | <0.001 | 0.82 | 0.64, 1.06 | 0.14 |
| HDL-C | 1,816 | 0.19 | 0.12, 0.28 | <0.001 | 0.64 | 0.25, 1.67 | 0.4 |
| LDL-C | 1,816 | 0.96 | 0.86, 1.07 | 0.4 | 0.63 | 0.33, 1.19 | 0.2 |
| non-HDL-C | 1,816 | 1.02 | 0.93, 1.11 | 0.7 |  |  |  |
| AIP | 1,816 | 3.49 | 2.47, 4.93 | <0.001 | 4.32 | 1.26, 14.8 | **0.020** |
| FPG | 1,816 | 1.06 | 1.04, 1.08 | <0.001 | 1.03 | 1.01, 1.05 | **0.014** |
| TnI | 1,816 | 1.01 | 1.00, 1.01 | 0.043 | 1.00 | 0.99, 1.01 | >0.9 |
| CK-MB | 1,816 | 1.00 | 1.00, 1.00 | 0.3 |  |  |  |
| NT-proBNP | 1,816 | 1.00 | 1.00, 1.00 | <0.001 | 1.00 | 1.00, 1.00 | 0.8 |
| LVEF | 1,816 | 0.97 | 0.96, 0.98 | <0.001 | 0.99 | 0.97, 1.00 | **0.020** |
| Antiplatelet drugs | 1,816 | 1.16 | 0.79, 1.69 | 0.4 |  |  |  |
| Statin | 1,816 | 0.91 | 0.64, 1.29 | 0.6 |  |  |  |
| Beta blocker | 1,816 | 1.35 | 1.09, 1.66 | 0.005 | 1.17 | 0.94, 1.46 | 0.15 |
| ACEI or ARB | 1,816 | 0.58 | 0.45, 0.74 | <0.001 | 0.77 | 0.58, 1.01 | 0.058 |
| Oral hypoglycemic agents | 1,816 | 1.30 | 1.04, 1.62 | 0.021 | 0.74 | 0.56, 0.99 | **0.039** |
| CCB | 1,816 | 1.05 | 0.86, 1.29 | 0.6 |  |  |  |
| Insulin therapy | 1,816 | 0.96 | 0.77, 1.20 | 0.7 |  |  |  |
| Dialysis therapy | 1,816 | 2.18 | 1.25, 3.78 | 0.006 | 0.89 | 0.48, 1.66 | 0.7 |

HR, Hazard Ratio; CI, confidence interval; MACCE, major adverse cardiac and cerebrovascular events; MI, myocardial infarction; AF, atrial fibrillation; CKD, chronic kidney disease; ACS, acute coronary syndrome; PCI, percutaneous coronary intervention; SBP, systolic blood pressure; DBP, diastolic blood pressure; HR, heart rate; LVEF, left ventricular ejection fraction; Hb, hemoglobin; PLT, platelet; Urea, carbamide; Scr, serum creatinine; eGFR, estimated glomerular filtration rate; UA, uric acid; TC, total cholesterol; TG, triglycerides; HDL-C, high-density lipoprotein-cholesterol; LDL-C, low-density lipoprotein-cholesterol; FPG, fasting plasma glucose; TnI, troponin I; CK-MB, creatine kinase-MB; NT-proBNP, N-terminal pro-brain natriuretic peptide; AIP, atherogenic index of plasma; ACEI, angiotensin-converting enzyme inhibitors; ARB, angiotensin receptor blockers; CCB, calcium channel blocker.

**Supplementary Table 2.** Distribution of sample size and MACCE events according to combined CKD strata in the overall cohort

| CKD subgroup | Total | MACCE | Non-MACCE |
| --- | --- | --- | --- |
| Stage 2–3 | 1,457 | 268 | 1,189 |
| Stage 4–5 | 359 | 111 | 248 |

MACCE, major adverse cardiac and cerebrovascular events; CKD, chronic kidney disease.

**Supplementary Table 3.** Sensitivity analysis of the association between elevated AIP and MACCE in Stage 2–3 versus Stage 4–5 CKD in the overall cohort

| CKD subgroup | N | Events | Adjusted HR (95% CI) | *P* value | *P* for interaction |
| --- | --- | --- | --- | --- | --- |
| Stage 2–3 CKD | 1,457 | 268 | 1.98 (1.47–2.66) | <0.001 | 0.149 |
| Stage 4–5 CKD | 359 | 111 | 2.92 (1.68–5.08) | <0.001 |  |

HR, hazard ratio; CI, confidence interval; MACCE, major adverse cardiac and cerebrovascular events; CKD, chronic kidney disease.

Adjusted HRs were derived from multivariable Cox proportional hazards models controlling for sex, age, previous myocardial infarction, hypertension, diabetes mellitus, hyperlipidemia, stroke history, smoking, anemia, myocardial infarction type, PCI, systolic blood pressure, heart rate, hemoglobin, urea, uric acid, LDL-C, non-HDL-C, fasting plasma glucose, troponin I, NT-proBNP, left ventricular ejection fraction, antiplatelet therapy, statin use, beta-blocker use, ACEI/ARB use, oral hypoglycemic agents, and dialysis.

**Supplementary Table 4.** Ordinal interaction analysis treating CKD stage as an ordered variable in the overall cohort

| Analysis | Interaction HR (95% CI) | *P* for interaction |
| --- | --- | --- |
| Ordinal CKD-stage interaction in the overall cohort | 1.08 (0.87–1.34) | 0.481 |

HR, hazard ratio; CI, confidence interval; CKD, chronic kidney disease.

**Supplementary Table 5.** Repeated advanced CKD subgroup sensitivity analysis after excluding patients who experienced MACCE within 30 days

| CKD subgroup | N | Events | Adjusted HR (95% CI) | *P* value | *P* for interaction |
| --- | --- | --- | --- | --- | --- |
| Stage 2–3 CKD | 1,417 | 228 | 1.46 (1.06–2.01) | 0.019 | 0.110 |
| Stage 4–5 CKD | 339 | 91 | 2.53 (1.41–4.54) | 0.002 |  |

HR, hazard ratio; CI, confidence interval; MACCE, major adverse cardiac and cerebrovascular events; CKD, chronic kidney disease.

Adjusted HRs were derived from multivariable Cox proportional hazards models controlling for sex, age, previous myocardial infarction, hypertension, diabetes mellitus, hyperlipidemia, stroke history, smoking, anemia, myocardial infarction type, PCI, systolic blood pressure, heart rate, hemoglobin, urea, uric acid, LDL-C, non-HDL-C, fasting plasma glucose, troponin I, NT-proBNP, left ventricular ejection fraction, antiplatelet therapy, statin use, beta-blocker use, ACEI/ARB use, oral hypoglycemic agents, and dialysis.

**Supplementary Table 6.** Ordinal CKD-stage interaction analysis after excluding patients who experienced MACCE within 30 days

| Analysis | Interaction HR (95% CI) | *P* for interaction |
| --- | --- | --- |
| Ordinal CKD-stage interaction in the overall cohort | 1.11 (0.89–1.38) | 0.359 |

HR, hazard ratio; CI, confidence interval; MACCE, major adverse cardiac and cerebrovascular events; CKD, chronic kidney disease.

**Supplementary Table 7.** Cox regression models for the associations between AIP and MACCE (sensitivity analysis).

| **Characteristic** | **Univariable** | | | | **Multivariate** | | |
| --- | --- | --- | --- | --- | --- | --- | --- |
|  | **N** | **HR** | **95% CI** | ***P* value** | **HR** | **95% CI** | ***P* value** |
| Sex | 1,713 | 0.72 | 0.57, 0.90 | 0.004 | 0.68 | 0.51, 0.89 | **0.006** |
| Age | 1,713 | 1.00 | 0.99, 1.01 | 0.7 | 1.02 | 1.01, 1.03 | **0.004** |
| Previous MI | 1,713 | 1.77 | 1.36, 2.31 | <0.001 | 1.43 | 1.08, 1.91 | **0.013** |
| Previous AF | 1,713 | 1.27 | 0.86, 1.87 | 0.2 |  |  |  |
| Hypertension | 1,713 | 1.59 | 1.11, 2.29 | 0.011 | 1.39 | 0.96, 2.02 | **0.085** |
| Diabetes | 1,713 | 2.05 | 1.64, 2.55 | <0.001 | 1.66 | 1.23, 2.22 | **<0.001** |
| Hyperlipidemia | 1,713 | 1.04 | 0.76, 1.42 | 0.8 | 1.05 | 0.76, 1.45 | 0.8 |
| Stroke History | 1,713 | 1.35 | 1.05, 1.73 | 0.020 | 1.20 | 0.92, 1.56 | 0.2 |
| Peripheral arterial disease | 1,713 | 1.15 | 0.59, 2.22 | 0.7 |  |  |  |
| CKD Stage | 1,713 |  |  |  |  |  |  |
| G2 |  | — | — |  | — | — |  |
| G3 |  | 1.94 | 1.49, 2.54 | <0.001 | 1.11 | 0.64, 1.91 | 0.7 |
| G4 |  | 2.40 | 1.60, 3.61 | <0.001 | 0.90 | 0.36, 2.25 | 0.8 |
| G5 |  | 2.56 | 1.90, 3.44 | <0.001 | 1.58 | 0.49, 5.08 | 0.4 |
| Anemia | 1,713 | 2.53 | 1.66, 3.88 | <0.001 | 1.16 | 0.71, 1.89 | 0.5 |
| Smoking | 1,713 | 1.09 | 0.86, 1.39 | 0.5 | 0.98 | 0.76, 1.28 | >0.9 |
| Drinking | 1,713 | 0.95 | 0.71, 1.26 | 0.7 |  |  |  |
| MI | 1,713 | 1.72 | 1.38, 2.14 | <0.001 | 1.40 | 1.07, 1.84 | **0.015** |
| PCI | 1,713 | 1.49 | 1.14, 1.95 | 0.004 | 1.15 | 0.87, 1.53 | 0.3 |
| SBP | 1,713 | 1.00 | 1.00, 1.01 | 0.6 | 1.00 | 0.99, 1.00 | 0.4 |
| DBP | 1,713 | 1.00 | 0.99, 1.01 | 0.5 |  |  |  |
| HR | 1,713 | 1.01 | 1.00, 1.01 | 0.10 | 1.00 | 0.99, 1.00 | 0.2 |
| Hb | 1,713 | 0.99 | 0.98, 0.99 | <0.001 | 0.99 | 0.99, 1.00 | 0.058 |
| PLT | 1,713 | 1.00 | 1.00, 1.00 | 0.7 |  |  |  |
| Urea | 1,713 | 1.06 | 1.05, 1.08 | <0.001 | 1.01 | 0.99, 1.04 | 0.4 |
| Scr | 1,713 | 1.00 | 1.00, 1.00 | 0.5 | 1.00 | 1.00, 1.00 | 0.2 |
| eGFR | 1,713 | 0.99 | 0.98, 0.99 | <0.001 | 0.99 | 0.98, 1.01 | 0.4 |
| UA | 1,713 | 1.00 | 1.00, 1.00 | 0.051 | 1.00 | 1.00, 1.00 | 0.8 |
| TC | 1,713 | 0.91 | 0.82, 1.00 | 0.059 |  |  |  |
| TG | 1,713 | 1.14 | 1.05, 1.23 | 0.002 |  |  |  |
| HDL-C | 1,713 | 0.25 | 0.16, 0.40 | <0.001 |  |  |  |
| LDL-C | 1,713 | 0.92 | 0.81, 1.03 | 0.2 | 0.73 | 0.42, 1.27 | 0.3 |
| non-HDL-C | 1,713 | 0.98 | 0.89, 1.08 | 0.7 | 1.26 | 0.78, 2.03 | 0.3 |
| **AIP** | 1,713 | 2.71 | 1.85, 3.96 | <0.001 | 1.98 | 1.16, 3.41 | **0.013** |
| FPG | 1,713 | 1.07 | 1.04, 1.09 | <0.001 | 1.04 | 1.01, 1.07 | **0.005** |
| TnI | 1,713 | 1.00 | 0.99, 1.01 | 0.9 | 0.99 | 0.98, 1.00 | 0.069 |
| CK-MB | 1,713 | 1.00 | 1.00, 1.00 | 0.7 |  |  |  |
| NT-proBNP | 1,713 | 1.00 | 1.00, 1.00 | <0.001 | 1.00 | 1.00, 1.00 | 0.6 |
| LVEF | 1,713 | 0.97 | 0.96, 0.98 | <0.001 | 0.98 | 0.97, 1.00 | **0.020** |
| Antiplatelet drugs | 1,713 | 1.12 | 0.75, 1.69 | 0.6 | 0.91 | 0.59, 1.38 | 0.6 |
| Statin | 1,713 | 1.08 | 0.72, 1.63 | 0.7 | 1.25 | 0.82, 1.91 | 0.3 |
| Beta blocker | 1,713 | 1.29 | 1.03, 1.62 | 0.027 | 1.17 | 0.92, 1.48 | 0.2 |
| ACEI or ARB | 1,713 | 0.61 | 0.47, 0.80 | <0.001 | 0.83 | 0.62, 1.11 | 0.2 |
| Oral hypoglycemic agents | 1,713 | 1.35 | 1.06, 1.72 | 0.014 | 0.71 | 0.52, 0.97 | **0.031** |
| CCB | 1,713 | 1.07 | 0.86, 1.33 | 0.6 |  |  |  |
| Insulin therapy | 1,713 | 0.82 | 0.64, 1.05 | 0.11 |  |  |  |
| Dialysis therapy | 1,713 | 2.51 | 1.41, 4.47 | 0.002 | 0.87 | 0.44, 1.71 | 0.7 |

HR, hazard ratio; CI, confidence interval; MACCE, major adverse cardiac and cerebrovascular events; MI, myocardial infarction; AF, atrial fibrillation; CKD, chronic kidney disease; ACS, acute coronary syndrome; PCI, percutaneous coronary intervention; SBP, systolic blood pressure; DBP, diastolic blood pressure; HR, heart rate; LVEF, left ventricular ejection fraction; Hb, hemoglobin; PLT, platelet; urea, carbamide; Scr, serum creatinine; eGFR, estimated glomerular filtration rate; UA, uric acid; TC, total cholesterol; TG, triglycerides; HDL-C, high-density lipoprotein cholesterol; LDL-C, low-density lipoprotein cholesterol; FPG, fasting plasma glucose; TnI, troponin I; CK-MB, creatine kinase-MB; NT-proBNP, N-terminal pro-brain natriuretic peptide; AIP, atherogenic index of plasma; ACEI, angiotensin-converting enzyme inhibitors; ARB, angiotensin receptor blockers; CCB, calcium channel blocker.
